# Factor structure and psychometric properties of the trier inventory for chronic stress (TICS) in a representative german sample

**DOI:** 10.1186/1471-2288-12-42

**Published:** 2012-04-01

**Authors:** Katja Petrowski, Sören Paul, Cornelia Albani, Elmar Brähler

**Affiliations:** 1 Dresden University of Technology, Department of Psychotherapy and Psychosomatic Medicine, Fetscherstrasse 74, Dresden D-01307, Germany; 2 University of Leipzig, Department of Medical Psychology and Medical Sociology, Philipp-Rosenthal-Strasse 55, Leipzig D-04103, Germany

**Keywords:** Chronic stress, Representative survey, Factor analysis, Measurement invariance

## Abstract

**Background:**

Chronic stress results from an imbalance of personal traits, resources and the demands placed upon an individual by social and occupational situations. This chronic stress can be measured using the Trier Inventory for Chronic Stress (TICS). Aims of the present study are to test the factorial structure of the TICS, report its psychometric properties, and evaluate the influence of gender and age on chronic stress.

**Methods:**

The TICS was answered by *N *= 2,339 healthy participants aged 14 to 99. The sample was selected by random-route sampling. Exploratory factor analyses with Oblimin-rotated Principal Axis extraction were calculated. Confirmatory factor analyses applying Robust Maximum Likelihood estimations (MLM) tested model fit and configural invariance as well as the measurement invariance for gender and age. Reliability estimations and effect sizes are reported.

**Results:**

In the exploratory factor analyses, both a two-factor and a nine-factor model emerged. Confirmatory factor analyses resulted in acceptable model fit (RMSEA), with model comparison fit statistics corroborating the superiority of the nine-factor model. Most factors were moderately to highly intercorrelated. Reliabilities were good to very good. Measurement invariance tests gave evidence for differential effects of gender and age on the factor structure. Furthermore, women and younger individuals, especially those aged 35 to 44, tended to report more chronic stress than men and older individuals.

**Conclusions:**

The proposed nine-factor structure could be factorially validated, results in good scale reliability, and heuristically can be grouped by two higher-order factors: "High Demands" and "Lack of Satisfaction". Age and gender represent differentiable and meaningful contributors to the perception of chronic stress.

## Background

Stress research indicates that chronic stress increases the risk of acute illness and impaired physical health [1,2]. Chronic stress has an influence on sleeping disorders [3], acute coronary syndrome [4], and chronic pain [5]. It follows that the conceptualization and the operationalization via instruments investigating chronic stress are of relevance to the psychology of health [6].

The concept of chronic stress is based on how frequently the stressors appear [7]. Chronic stress is defined as the repeated occurrence of different stressors with uncontrollable consequences or the absence of adaptive coping mechanisms [8,9]. In addition, it may evolve from an ongoing lack of satisfaction of an individual's needs, e.g. the need for appreciation, social support, meaningful tasks, and diversification [10] as well as from the conformity to social roles [11], or from *non-events*, i.e. desired events that do not occur such as pregnancy [12,13]. According to the systemic requirement - resource model of health [14], individual health might be promoted and preserved when high internal demands and external demands can be satisfied via employing the available internal or external resources [15]. Demands are claims that a person has to the environment based on values and needs, and towards itself based on individual strivings. Resources might be ecological, societal, occupational, or private nature. Psychological resources might be personality traits such as sense of coherence, self-efficacy, and competencies to apply resources appropriately. In sum, individual health depends on the fulfillment of needs based on resources, while chronic stress might stem from constantly low or high demands, or from a lack of resources.

Cohen and colleagues [2] provide a thorough overview of English assessment instruments, e.g. the Perceived Stress Scale [16]. However, the Trier Inventory for Chronic Stress (TICS) by Schulz, Schlotz, and Becker [17] is the first instrument that explicitly captures chronic psychosocial stress within nine factors: Work Overload, Social Overload, Pressure to Perform, Work Discontent, Excessive Demands from Work, Lack of Social Recognition, Social Tensions, Social Isolation, and Chronic Worrying. In accordance with the systemic requirement - resource model of health [14], these nine factors can be grouped into High Demands referring to specific job conditions and social conditions, and Lack of Satisfaction of one's needs due to unsatisfactory job conditions and social conditions (see [17], pp. 38-39).

Since the TICS scales were developed based on the systemic requirement - resource model of health [14], the authors postulated content validity as a logical consequence [17] with factorial validity being more of a concern. The factorial structure was changed three times: from 39 items on six factors [18] to 62 items on ten factors [19] and, lastly, to the present 57 items on nine factors [17]. Calculations of the first two versions had been based on non-representative ad-hoc samples of *N *= 157 first-semester psychology students [18] and a mixture of fitness-studio users, sociology students, social workers, nurses, and others totaling *N *= 815 (see [19], pp. 16-17). The recent version had been constructed using a sample of *N *= 604 adults aged 16 to 70 who were randomly selected from telephone registers after calculating representative numbers of participants from statistics from the German Federal Statistical Office for the year 1999 (see [17], p. 51).

Although the factor-analytical analyses for the most recent version showed good factorial validity, it remains unclear whether it can be replicated using a representative sample. Consequently, one of the aims of the present study was to test the factorial structure of the TICS by confirmatory factor analyses using a representative sample. The TICS authors found a nine-factor model based on the item values and a two-factor model based on the sum scores of the nine scales [17]. Thus, it can be hypothesized that based on item values the nine-factor model yields a better model fit to empirical data than the two-factor model. Furthermore, the superior factor structure was tested for measurement invariance between the genders and among the age groups. Based on the literature [17] it could be postulated that women report more stress than men and younger persons report more stress than older ones. However, it is not yet clear whether the characteristics of the genders and the age groups differ also in respect to the factors postulated to be underlying chronic stress. Recent findings indicate that measurement invariance may be absent [20], i.e. the latent structure of constructs, factors, and items might differ between different groups, e.g. between men and women. According to previous data [17], reliability based on the representative sample may be good to very good.

## Methods

### Sample

The data collection was conducted by the 'USUMA' polling institute in Berlin, Germany, on behalf of the University of Leipzig in 2004. The participants filled out the questionnaires in their homes. Households and participants were selected by the random-route sampling method. The random-route sampling method is characterized as national sample "based on a combination of random and systematic and stratified probability samples at different levels. First, a stratified selection of sampling units took place, then out of it a systematic selection of households by random walk and at the end a random selection of one person per household by Kish-table was administered." ([21], p. 206]). This sample was then validated based on information obtained from the German Federal Statistical Office [22]. The coverage rate was 62.3%, resulting in the basic sample of *N *= 2,473 participants. The exclusion of non-native German speakers and incomplete data sets resulted in the acceptable loss of only *n *= 134 cases (5.4% of all cases) and the final sample of *N *= 2,339 cases with a mean age of *M *= 47.9 (*SD *= 17.92, range = 14-99), including 52.6% female participants. Other socio-demographic details of the basic sample, including those participants who had not completed the questionnaire, have been reported elsewhere [23]. All the participants volunteered and received a data protection declaration that is in agreement with the Helsinki Declaration. The study was approved according to the ethical guidelines of the "German Professional Institutions for Social Research" [Arbeitskreis Deutscher Markt- und Sozialforschungsinstitute, Arbeitsgemeinschaft Sozialwissenschaftlicher Institute, Berufsverband Deutscher Markt- und Sozialforscher].

### Instruments

The *Trier Inventory for Chronic Stress *(TICS) [17] is a standardized questionnaire for assessing nine interrelated factors of chronic stress: Work Overload (e.g. "I have too many tasks to perform."), Social Overload (e.g. "I must frequently care for the well-being of others."), Pressure to Perform (e.g. "I have tasks to fulfill that pressure me to prove myself."), Work Discontent (e.g. "Times when none of my tasks seem meaningful to me."), Excessive Demands at Work (e.g. "Although I try, I do not fulfill my duties as I should."), Lack of Social Recognition (e.g. "Although I do my best, my work is not appreciated."), Social Tensions (e.g. "I have unnecessary conflicts with others."), Social Isolation (e.g. "Times when I have too little contact with other people."), and Chronic Worrying (e.g. "Times when I worry a lot and cannot stop."; all the items were translated by W. Schlotz and P. Schulz, personal communication, October 23, 2011). The participants rated all the 57 items on a five-point Likert scale in respect to how often they have experienced a certain situation or have had a certain experience within the last three months (0 = never, 1 = rarely, 2 = sometimes, 3 = often, 4 = very often). Internal consistency (Cronbach's Alpha) as reported for the original samples by Schulz, Schlotz, and Becker [17] with a mean of α = .87 and a range from .84 to .91 indicates good to very good reliability.

### Statistical procedure

All item and exploratory factor analyses were carried out using SPSS 16.0 and LISREL 8.80s/PRELIS 2.80s. Since conducting both the exploratory factor analyses (EFA) and the confirmatory factor analyses (CFA) using the same sample would lead to artificially increased model fit values, the sample was randomly divided into two partial samples, one for each procedure (*n*_EFA _= 1,190, *n*_CFA _= 1,149). No statistically significant differences were found through an ANOVA corrected for unequal variances between the two samples for all the TICS items and the socio-demographic variables with all *F*_Brown-Forsyth _(1, 2066) < 3.00 (all *p *> .083).

Several EFAs were conducted by applying the same extraction and rotation methods formerly described by the TICS authors (see [17], p. 38), i.e. the Principal Axis extraction method in order to adjust for non-normal item distributions [24], and the oblique Oblimin-rotation as all items tended to measure stress in related stress domains. Based on recommendations by Brown [24] and Gorsuch [25], the Oblimin power parameter δ was systematically altered between user-defined values of -10, -5, -1, and 0 to 0.8 to find the solution with the least number of complex items, i.e. items with cross-loadings of > .30 on more than one factor, and hyperplane items, i.e. items without loadings of > .30 on any factor.

Using Mplus 5.1, the respective fit of the two-factor and the nine-factor model were tested using CFAs. The Robust Maximum Likelihood estimation (MLM) with standard errors and a mean-adjusted chi-square test statistic, the Satorra-Bentler chi-square (SB χ^2^), were used in order to account for the non-normality of the data [24]. According to Schermelleh-Engel, Moosbrugger, and Müller [26], good (acceptable) model fit is a given with SB χ^2^/df index below 2.0 (below 3.0), Comparative Fit Index (CFI) as well as Tucker-Lewis-Index (TLI) above .95 (above .90), Standardized Root Mean Square Residual (SRMR) below .05 (below .10), and Root Mean Square Error of Approximation (RMSEA) below .05 (below .08). The model comparison was established by choosing the model with the lowest Akaike Information Criterion (AIC), the lowest values in the scaled difference of chi-squares test according to Satorra and Bentler (SDCS test, see [24], pp. 385-387), and by reporting the descriptive fit indices.

For the ANOVAs testing for differences between genders (women vs. men) or age groups (14-34 years old vs. 35-44 years vs. 45-99 years), effect sizes of η^2 ^> .01 are considered to be weak, of η^2 ^> .09 to be moderate, and η^2 ^> .25 to be strong according to Cohen [27].

## Results

### Descriptive item analysis

Significant univariate non-normality with *p *< .05 was shown regarding skewness (all but one item), kurtosis (50 of the 57 items), and in the Shapiro-Wilk test of non-normality with *W *> .81 (*p *< .001). Further, significant multivariate non-normality was found with Mardia's multivariate skewness (β_1, *p *_= 227.3, χ^2 ^= 88,603.3, *p *< .001) and Mardia's multivariate kurtosis (β_2, *p *_= 4,307.8, *n *(β_2, *p*_) = 278.6, *p *< .001) [28].

### Exploratory factor analysis (EFA)

The Scree test pointed toward a solution with two factors that resemble the High Demands factor and the Lack of Satisfaction factor described by Schulz, Schlotz, and Becker [17]. The Kaiser-Guttman criterion provided evidence for a model with seven to nine factors, EFA eigenvalues were 21.73, 4.96, 2.06, 1.49, 1.40, 1.24, 1.15, 1.00, 0.94, and 0.88. To our knowledge, the traditional 1.00 cut-off is more of a rule-of-thumb and there is no established confidence interval around it. Thus, the determination of the factor number also needs to be based on theoretical assumptions and the Kaiser-Guttman criterion that can, in this case, be interpreted as pointing towards the published solution with the nine factors [17]. Testing the seven and eight factors model did not result in further evidence for or against the proposed nine-factor model.

The item assignments and the communalities of the emerged models can be seen in Table 1. The two-factor model with δ = 0.5 resulted in the simplest structure with no hyperplane items, nine complex items, and two items found in a factor other than the one expected. The two factors explained 17% of the variance, respectively, and are highly and positively intercorrelated with *r*_12 _= .56. The nine-factor model with δ = -1.0 resulted in the simplest structure with three hyper-plane items, eight complex items, and two items found in a factor other than the one expected. Factor six, equivalent to Lack of Social Recognition, produced most of these item assignment problems regardless of the δ value. The variance explained by each factor can be seen in Table 1. Factor intercorrelations were significant and, almost always, small to moderate with *r*_ij _= .13 to .46, all *p *< .05, *M *= .30, *SD *= .0092. For factor two, representing the Social Isolation scale, the intercorrelations *r_23 _*= .04, *r_27 _*= .03, and *r_29 _*= .04 were not significant.

**Table 1:** Exploratory factor analyses: factor matrix (Principal Axis extraction, Oblimin rotation, δ = 0.5 for two-factor solution, δ = - 1.0 for nine-factor solution, n *= 1,190)*

TICS original factor	Two-factor solution			Nine-factor solution									
**Item**	***h*^2^**	**Factor 1**	**Factor 2**	***h*^2^**	**Factor 1**	**Factor 2**	**Factor 3**	**Factor 4**	**Factor 5**	**Factor 6**	**Factor 7**	**Factor 8**	**Factor 9**

*Work Overload*				11.0%									

01	.28	**.48**	.08	.41	-.02	.12	-.02	.08	-.13	**.40**	.21	.00	.19

04	.37	**.53**	.12	.51	.04	.01	-.01	-.03	.01	**.42**	**.33**	.15	.03

17	.50	**.72**	-.01	.62	.07	-.02	.12	.15	-.05	.17	**.57**	-.03	.04

27	.55	**.80**	-.11	.65	.07	.00	.19	.00	-.03	.05	**.60**	.02	.12

38	.54	**.66**	.11	.62	.10	.00	.09	.07	.13	.00	**.50**	.09	.16

44	.53	**.64**	.15	.64	.10	.07	.06	.12	-.09	.10	**.52**	.16	.10

50 $	.49	**.70**	.00	.60	.00	.07	.04	-.06	.03	.13	**.52**	.10	.22

54	.51	**.46**	**.35**	.63	-.02	.10	-.05	.05	.11	.08	**.34**	**.37**	.21

*Social Overload*				6.9%									

07	.49	**.82**	-.28	.57	.08	-.10	**.31**	.02	-.11	.23	.02	-.06	**.41**

19	.50	**.65**	.10	.53	.11	-.12	.08	.19	.06	.18	.15	.10	.29

28	.50	**.64**	.11	.55	.12	-.03	.03	.20	.04	.05	.24	.05	**.38**

39 $	.44	**.73**	-.12	.59	.03	-.06	.10	.11	.06	.03	.07	-.05	**.63**

49	.44	**.77**	-.24	.58	-.05	-.02	.27	-.02	.00	.10	.00	.02	**.58**

57	.50	**.46**	**.34**	.59	.14	.00	-.12	.10	.14	.07	.21	.28	**.31**

*Pressure to Perform*				7.8%									

08	.45	**.68**	-.02	.58	.08	-.06	**.51**	.16	.00	.19	-.03	.00	.15

12	.36	**.36**	**.32**	.45	.10	.15	**.37**	.16	.12	.17	.00	-.03	.00

14	.48	**.67**	.04	.54	.21	-.05	**.45**	-.03	.08	.09	.15	.11	.03

22	.48	**.76**	-.13	.60	-.03	-.01	**.58**	.00	.05	.05	.08	.08	.21

23	.36	**.65**	-.10	.54	-.04	.03	**.65**	.03	.03	.00	.09	.05	.08

30	.37	**.48**	.19	.43	.14	.16	**.32**	.16	-.03	.06	.09	-.03	.16

32 $	.48	**.75**	-.13	.60	.03	.01	**.59**	-.07	.03	.01	.10	.11	.18

40	.43	**.51**	.21	.52	.15	.03	**.38**	.14	.19	-.06	.01	.04	.21

43 #	.58	**.85**	-.20	.66	.03	.04	**.44**	.03	-.08	.00	.23	-.01	**.35**

*Work Discontent*				10.2%									

05	.36	**.44**	.23	.55	.16	-.12	.08	-.02	**.38**	.25	**.32**	.01	-.12

10	.35	-.17	**.67**	.46	.01	.19	-.02	.18	**.47**	.11	-.14	-.02	-.02

13	.34	**.44**	.20	.47	.13	-.10	.19	.06	**.37**	.13	.28	-.05	-.09

21 $	.43	-.07	**.69**	.54	.00	.18	.08	.16	**.49**	.06	-.07	.10	-.07

37	.45	**.37**	**.39**	.55	.23	-.06	.02	.13	**.31**	-.09	**.31**	.15	.04

41	.43	-.23	**.75**	.52	.09	.28	.05	.16	**.42**	-.16	-.07	.09	-.06

48	.33	.28	**.37**	.46	.05	.02	-.01	-.06	**.46**	.16	.01	.13	.21

53	.45	-.02	**.68**	.55	.04	**.31**	-.02	.10	**.46**	-.01	.01	.03	.14

*Excessive Demands at Work*				8.3%									

03	.39	.24	**.46**	.52	.05	.06	.06	.08	.05	**.41**	.00	**.35**	-.08

20	.45	.22	**.52**	.58	.09	-.01	.12	.15	.04	.15	.01	**.53**	-.10

24	.44	.17	**.55**	.60	.13	.04	.05	.12	-.01	.05	-.04	**.62**	.01

35	.50	.18	**.59**	.58	.14	.09	.13	.19	.07	-.04	.05	**.47**	-.03

47	.38	.15	**.52**	.47	-.05	.15	-.02	.15	.05	.06	.13	**.44**	.05

55 $	.48	.22	**.55**	.62	.07	.10	.02	.05	.09	.00	.09	**.59**	.07

*Lack of Social Recognition*				9.0%									

02	.32	**.31**	**.33**	.49	.08	.05	-.05	.07	.15	**.53**	-.06	.03	.13

18	.43	**.32**	**.42**	.47	.16	.00	.10	.07	.17	.24	.03	.25	.00

31 $	.44	.25	**.49**	.48	.21	.09	.02	.00	.17	.14	.00	**.33**	.11

46	.44	**.39**	**.36**	.51	.20	.00	.01	-.05	.27	.14	.08	.25	.18

*Social Tensions*				8.1%									

06	.44	**.38**	**.37**	.57	**.49**	.00	.09	.07	.12	.26	.01	-.03	.02

15	.39	**.30**	**.40**	.59	**.63**	-.03	.08	.10	.02	.18	.00	-.01	-.04

26	.46	.21	**.54**	.61	**.59**	.04	-.01	.15	.05	.00	.02	.11	.03

33	.49	**.30**	**.49**	.56	**.48**	.11	.09	.07	.06	.02	.11	.12	.02

45	.53	.25	**.56**	.67	**.58**	.10	-.02	.14	.00	-.08	.12	.16	.07

52 $	.44	.16	**.56**	.59	**.59**	.18	-.08	.00	.07	.01	.06	.08	.08

*Social Isolation*				10.1%									

11	.41	-.29	**.75**	.55	-.05	**.63**	.05	.15	.05	.12	-.03	.01	-.12

29	.46	.14	**.59**	.50	.13	**.40**	.07	.16	.03	.09	.07	.07	.11

34	.53	-.14	**.79**	.58	.21	**.50**	.01	.17	.01	-.03	.01	.14	-.02

42 $,#	.53	**-.30**	**.85**	.67	.09	**.68**	.03	.10	.09	-.03	.02	.05	-.07

51	.53	-.28	**.85**	.70	.07	**.75**	-.02	.01	.07	-.01	.03	.10	.00

56	.43	-.14	**.72**	.51	.04	**.53**	-.05	.12	.13	.07	.00	.02	.09

*Chronic Worrying*				8.1%									

09	.35	.17	**.48**	.46	.11	.07	.09	**.44**	.05	.21	-.08	.00	.03

16	.39	.08	**.57**	.59	.06	.11	.00	**.63**	-.02	.18	-.02	-.04	.01

25	.47	.12	**.61**	.58	.02	.04	-.03	**.55**	.07	.03	.07	.21	.03

36 $	.49	.07	**.66**	.68	.04	.08	-.04	**.68**	.05	-.08	.08	.13	.07

Item enumeration, item assignment, and marker items for the two-factor model (#) and the nine-factor model ($) are according to Schulz, Schlotz, and Becker [17]. *h*^2 ^= explained variance by the respective factor or item communalities, i.e. percentage of item variance explained by the latent factor. Boldfaced factor loadings are above the .30 threshold

### Confirmatory factor analysis (CFA)

Item assignments were adopted from Schulz, Schlotz, and Becker (see [17], p. 29 and p. 35) and can be seen in the first column of Table 1. Complex items of this solution, i.e. items 27, 49, and 54, were assigned to all the factors on which they had a salient loading. The items with the highest salient loading on their respective factor were chosen to be marker indicators. In order to maximize the simplicity of the structure, indicator errors were specified as random and uncorrelated.

#### Configural invariance

To test for configural invariance, models with two and nine factors were compared to each other. Based on the given recommendations [26], the overall fit statistics in Table 2 suggest unacceptable absolute fit (SB χ^2^/*df*) and unacceptable comparative fit according to the TLI and CFI, but also acceptable comparative fit according to the SRMR and acceptable parsimony fit (RMSEA) for each model. Both the RMSEA and the TLI correct for parsimony, which is essential in judging a model with lots of indicators, however, only the RMSEA provides a test against the perfect model. Two of the three criteria for reasonably good fit according to Hu and Bentler [29] are met, i.e. SRMR below .08 and RMSEA close to .06 or below. Thus, overall model fit might be cautiously interpreted as acceptable. Both the AIC and the RMSEA indicate the superiority of the nine-factor model.

**Table 2:** Confirmatory factor analysis: Model comparison and tests of measurement invariance for the nine-factor model (Robust Maximum Likelihood estimation - MLM)

Model tested			Absolute fit			Comparison to less-constrained (or baseline) model		Comparative fit				Parsimony fit
	***N***	**c_1_**	**SB χ^2^**	***df***	**SB χ^2^/*df***	**Δ*df***	***T_S _*(ΔSB χ^2^)**	**AIC**	**TLI**	**CFI**	**SRMR**	**RMSEA ^a^**

**Factorial Invariance**												

Two-factor model	1,149	1.259	11,847.949***	1,538	7.7			157,955	.747	.756	.078	.067

Nine-factor model	1,149	1.250	7,383.383***	1,500	4.9	38	2,765.7***	153,564	.855	.863	.071	.051

**Measurement Invariance (Gender)**												

Female	622	1.209	4,837.946***	1,500	3.2			82,414	.858	.867	.071	.052

Male	527	1.220	4,720.306***	1,500	3.1			71,065	.830	.840	.077	.055

Baseline Model	1,149	1.211	9,665.630***	3,048	3.2			153,488	.846	.853	.075	.053

Partial measurement invariance ^b^	1,149	1.209	9,761.882***	3,099	3.2	51	88.4**	153,482	.847	.852	.077	.053

Full measurement invariance ^c^	1,149	1.210	9,874.099***	3,157	3.1	58 (109)	88.8*** (176.4***)	153,482	.849	.851	.078	.053

**Measurement Invariance (Age)**												

Young (14-47)	540	1.193	4,562.580***	1,500	3.0			73,432	.834	.844	.075	.054

Old (48-99)	609	1.251	4,743.958***	1,500	3.2			79,730	.863	.871	.068	.050

Baseline Model	1,149	1.219	9,481.756***	3,048	3.1			153,245	.849	.856	.073	.052

Partial measurement invariance ^b^	1,149	1.215	9,571.958***	3,099	3.1	51	92.4***	153,236	.850	.854	.075	.052

Full measurement invariance ^c^	1,149	1.217	9,812.218***	3,157	3.1	58 (109)	181.5*** (248.6***)	153,358	.849	.850	.076	.052

Measurement invariance tested for the nine-factor structure. c_1 _= χ^2 ^scale correction factor for Robust Maximum Likelihood estimation (MLM). SB χ^2 ^= Satorra-Bentler scaled χ^2 ^value. *T_S _*(ΔSB χ^2^) = test statistic for the scaled difference in SB χ^2^s (SDCS test) with *df *as stated above comparing a model with the model above. ** *p *< .01, *** *p *< .001. AIC = Akaike information criterion. **^a ^**RMSEA 90% confidence interval is not available for MLM estimations in MPlus 5.1. ^b ^Factor loadings and indicator intercepts constrained as equal. ^C ^Factor loadings, indicator intercepts, and indicator errors hold equal

#### Measurement invariance

To test the measurement invariance of the approved nine-factor model, several multiple-group CFAs were conducted contrasting women and men as well as younger participants aged 14 to 44 years and older participants aged 45 to 99. As can be seen in Table 2 the CFAs did not support measurement invariance for either gender or age. Although models with decreasing number of freely estimated parameters did not markedly differ in their RMSEA, their CFI and SB χ^2 ^differed significantly from the less restrictive model and, obviously, also from the baseline model. Hence, the factor model may not be equivalent by gender and by age.

### Effects of gender and age

The analyses of variance were conducted for each scale of the nine-factor model with the factors gender and age group (cf. Table 3). Compared to men, women reported significantly more chronic stress for the scales Social Overload, Pressure to Perform, Social Isolation, and Chronic Worrying with all *F*(1, 2336) > 8.326, all *p *< .01, however, the effects were weak or meaningless as indicated by η^2 ^= .004 to .007. As for age, most scales show a peak in younger participants, especially those aged 35-44, and continuously drop from thereon. Except for Social Isolation, the overall differences between the age groups were significant with all *F*(2, 2335) > 3.971, all *p *< .05, η^2 ^= .003 to .098. Post-hoc Tukey tests were undertaken, revealing the results as stated in Table 3.

**Table 3:** TICS scale properties for different age cohorts and for gender

Scale	Items	Range ^a^	Reliabilities			Skewness	Kurtosis	*M (SD)*					
			**Cronbach's α**	**Split-half ^b^**	**ISC range**			**Total sample**	**14-44 years**	**45-64 years**	**65-99 years**	**Women**	**Men**

								*N *= 2,339	*N *= 1,087	*N *= 746	*N *= 506	*N *= 1,231	*N *= 1,108

Work Overload	8	0-31(0-32)	.88	.87	.52-.74	.24***	-.48***	9.8 (6.22)	11.4*** (5.85)	9.8*** (6.11)	6.4 (5.74)	10.0 (6.28)	9.6 (6.16)

Social Overload	6	0-24(0-24)	.86	.87	.57-.69	.27***	-.48***	7.5 (4.97)	8.4** (4.82)	7.6*** (4.78)	5.2 (4.89)	7.8** (5.10)	7.1 (4.81)

Pressure to Perform	9	0-35(0-36)	.90	.90	.58-.72	.09	-.53***	12.3 (7.10)	14.2*** (6.65)	12.1*** (6.89)	8.5 (6.78)	11.9** (7.07)	12.7 (7.11)

Work Discontent	8	0-32(0-32)	.85	.85	.53-.66	.25***	-.30**	9.7 (5.55)	10.3 (5.49)	9.7*** (5.55)	8.1 (5.35)	9.6 (5.58)	9.7 (5.51)

Excessive Demands at Work	6	0-24(0-24)	.87	.87	.61-.71	.63***	-.06	5.6 (4.28)	5.9 (4.29)	5.5 (4.13)	5.1 (4.43)	5.7 (4.30)	5.5 (4.26)

Lack of Social Recognition	4	0-16(0-16)	.81	.82	.58-.67	.42***	-.39***	4.5 (3.21)	5.0** (3.19)	4.6*** (3.10)	3.3 (3.10)	4.5 (3.22)	4.5 (3.20)

Social Tensions	6	0-24(0-24)	.88	.88	.65-.73	.56***	-.11	6.1 (4.55)	6.8** (4.41)	6.1*** (4.48)	4.7 (4.63)	6.0 (4.54)	6.2 (4.56)

Social Isolation	6	0-24(0-24)	.82	.81	.56-.71	.59***	-.07	4.6 (3.41)	6.9 (4.84)	7.3 (4.98)	7.3 (5.43)	7.5*** (5.14)	6.7 (4.84)

Chronic Worrying	4	0-16(0-16)	.83	.84	.58-.71	.53***	-.06	4.8 (3.34)	4.9 (3.35)	4.9* (3.32)	4.4 (3.34)	5.0*** (3.39)	4.5 (3.27)

Chronic Stress Screening Scale	12	0-46(0-48)	.91	.88	.55-.71	.33***	-.48***	13.3 (8.34)	14.4* (8.31)	13.4*** (8.08)	11.0 (8.34)	13.7* (8.46)	12.9 (8.18)

^a ^theoretical range in brackets. ^b ^Split-half reliabilities adjusted after Spearman-Brown. ISC range = range of Item-scale correlations. Significances (tests of univariate non-normality, ANOVAs for age groups and gender): * *p *< .05, ** *p *< .01, *** *p *< .001, significances to the next-older age group are presented. For age groups, Tukey-HSD post-hoc tests are reported

### Reliability and descriptive scale analysis

In most cases, the nine TICS scales were moderately to highly intercorrelated with *r*_ij _= .30 to .77, *M *= .58, *SD *= .112, all *p *< .05, *N *= 2,339. As may be seen in Table 3 reliabilities were good to very good with the coefficient *α *and the adjusted split-half reliabilities ranging from .81 to .91. Significant non-normality was found regarding skewness of nine scales and regarding kurtosis within six scales. Most scales tended to be significantly left-skewed and flatter than the Gaussian distribution. Remarkably, 32-54% of the participants scored an average of < 1 on a certain scale (*M *= 40.9%, *SD *= 6.2%), including 5-13% reporting no certain stressful event on at least one scale (*M *= 9.4%, *SD *= 3.1%).

## Discussion

One of the aims of the present study was to test the factorial structure of the TICS by means of confirmatory factor analyses using a representative sample. This included tests of measurement invariance for gender and age groups. Furthermore, the reliability of the resulting nine scales as well as the influences of gender and age on chronic stress were investigated.

### Nine factors of chronic stress heuristically grouped

The EFA produced evidence for two possible solutions that were tested during subsequent CFAs. The emerged two-factor structure closely resembled the two-factor solution which the TICS authors reported [17]. The High Demands factor resembled "stress which results from high demands in combination with specific job conditions and social conditions". The Lack of Satisfaction factor had been described as the "lack of satisfaction of one's needs due to unsatisfactory job conditions and social conditions", i.e. "lack of safety, appreciation, success, social contacts, and meaningful tasks" ([17], pp. 38-39, translated). While High Demands is supposed to be closely associated with age, job situation, and marital status, Lack of Satisfaction is said to be associated with personality traits such as neuroticism [17]. Both factors explained merely 17% of the variance in the empirical data. The two proposed factors could therefore be replicated on the item level but might better be interpreted as a heuristic grouping of the nine validated factors underlying chronic stress. Empirical results proved the two factors' importance in determining stress-related health impairment [30].

The nine-factor solution as proposed by the TICS authors [17] was supported by the EFA in factor number and item assignment, and the good to very good reliability of its scales was replicated in this representative sample. While both models resulted in an acceptable fit for the present data, the nine-factor model was shown to be the superior one.

The low CFI and TLI values might stem from the TICS' broad conception which covers nine different groups of stressful events. According to Marsh, Hau, and Wen [31], recommendations like those by Hu and Bentler [29] are hardly met by questionnaire data. This is especially true for questionnaires with many items, like the TICS that consists out of 57 items on nine scales. Furthermore, the authors argue that goodness-of-fit indices may be more appropriate for comparing models with each other instead of judging the model fit itself.

### Measurement invariance - differential influences of gender and age

The nine different groups of stressful events are supposed to be crucial contributors to chronic stress. However, their structure was shown to be non-invariant between genders and among age groups. Furthermore, the scale values are associated with the genders but only to a much lower degree with age. Possible gender differences might be attributed to the differential interaction of androgens and stress hormones which in turn affect the perception and the evaluation of aversive stimuli (for review, see [32]). Likewise, androgen and estrogen receptors are localized in brain regions involved in the control of endocrine responsiveness to stress. Preliminary animal studies found enhanced corticosteroid release in aversive situations depending on the acute administration of different steroids compared to no steroid administration, but not for the long-term administration [32]. As for age, the majority of events were reported by young and middle-aged participants, especially between the ages of 35 to 44, indicating that during this stage in life, the risk for chronic stress is the highest. Typically, individuals of this age-groups tend to be busy engineering a career parallel to seeking fulfillment in their private lives and dealing with the puberty of their offspring. Interestingly, comparable differences have been reported both for the TICS scale values [17] and for the factor structure of other questionnaires on perceived stress [20]. It remains an open question whether and why these differences appear, whether there might be more than just the psychoneuroendocrinological basis to explain them, and how researchers as well as practitioners can use the results to better understand chronic stress and its health implications.

In contrast to the age-related pattern described above, the Social Isolation scale values remain constant or even increase in older participants. While retirement may mean less stress due to a reduction in work-related interactions, tasks, and responsibilities, it may also reduce the number or the intensity of possible social contacts, self-esteem-enhancing interactions, and opportunities for success leading to the hypothesis that less work-related stress may not at all make up for the loss or lack of social resources and meaningful tasks.

The question remains, what other constructs besides the frequency of stressors (as measured by the TICS) mediate or moderate the accumulation of stressful, which in turn lead to chronic stress. Other possible constructs might be the interaction of the perceived intensity of the event, its consequences to (perceived and real) social, emotional, and financial coping resources, coping preferences [33], individual differences in stress reactivity [34], and earlier individual stress patterns that might produce vulnerabilities and protective factors [35,36]. Emotion regulation strategies seem to change with time resulting in increased conflict avoidance and emotional control [37,38]. Martin [39] reported that elderly people were especially at risk since they were subject to more stressful life events and less access to resources. In sum, life-stage-specific events, idiosyncratic events, coping strategies, resources, and differences in self-regulation might be subject to further elaboration for their incremental influence on reported stressful events accumulating to chronic stress as the TICS showed.

### Limitations

It is not possible to draw general conclusions based on the data from a representative sample as the large sample size could easily lead to significant effects. Since the sample was representative for the normal population, the results are not offhandedly applicable to highly stressed samples. In turn, the TICS should be applied to different professional groups and to clinical samples to further replicate or reprobate the factorial structure. As for now, the instrument is still lacking an English translation and validation, thus the presented factors, psychometrics, and effects may differ in non-German populations.

### Future research indications

It would be productive to test the stability of the chronic stress construct (test-retest reliability) and to explore connections to other chronic stress questionnaires (convergent validity) or external ratings (criterion validity). A design with repeated measurements would allow for the comparison of factor structures across time and determination of possible cohort effects.

## Conclusions

In sum, the factorial structure of the TICS could be replicated using several exploratory and confirmatory factor analyses. The nine-factor model generally reproduced the item assignment found by the TICS authors and resulted in a better model fit than other possible models. Furthermore, a heuristic model with two higher-order factors High Demands and Lack of Satisfaction could be explicated. The nine TICS scales showed good to very good reliability. Gender and age effects on chronic stress could be replicated. Women and younger individuals, especially those aged 35 to 44, tended to report more chronic stress than men and older individuals.

## Competing interests

The authors declare that they have no competing interests.

## Authors' contributions

EB and CA were responsible for the conception and the design of the study as well as the acquisition of the data. KP and SP performed the statistical analyses, contributed to the interpretation of the data, wrote the first and the final version of the manuscript, and critically revised the manuscript for intellectual content. All the authors read and approved the final version of the manuscript for publication.

## Pre-publication history

The pre-publication history for this paper can be accessed here:

http://www.biomedcentral.com/1471-2288/12/42/prepub
